# Validation of a preclinical model for assessment of drug efficacy in melanoma

**DOI:** 10.18632/oncotarget.7541

**Published:** 2016-02-20

**Authors:** Julie Delyon, Mariana Varna, Jean-Paul Feugeas, Aurélie Sadoux, Saliha Yahiaoui, Marie-Pierre Podgorniak, Geoffroy Leclert, Sarra Mazouz Dorval, Nicolas Dumaz, Anne Janin, Samia Mourah, Céleste Lebbé

**Affiliations:** ^1^ INSERM UMR_S976, Paris, F-75010, France; ^2^ AP-HP, Hôpital Saint-Louis, Department of Dermatology, Paris, F-75010, France; ^3^ Université Paris-Diderot, Sorbonne Paris Cité, Paris, F-75013, France; ^4^ INSERM UMR_S1165, Paris, F-75010, France; ^5^ Université Paris-Diderot, Department of Pathology, UMR_S1165, Paris, F-75010, France; ^6^ UMR CNRS 8612, Institut Galien-UFR de Pharmacie, Université de Paris-Sud, Châtenay-Malabry, F-92290, France; ^7^ INSERM UMR_1137, Paris, France; ^8^ AP-HP, Hôpital Saint-Louis, Laboratoire de Pharmacologie Biologique, Paris, F-75010, France; ^9^ AP-HP, Hôpital Saint-Louis, Department of Plastic, Reconstructive and Esthetic Surgery, Paris, F-75010, France; ^10^ AP-HP, Hôpital Saint-Louis, Department of Pathology, Paris, F-75010, France

**Keywords:** histoculture drug response assay, xenograft, BRAF mutation, melanoma, model

## Abstract

The aim of personalized medicine is to improve our understanding of the disease at molecular level and to optimize therapeutic management. In this context, we have developed *in vivo* and *ex vivo* preclinical strategies evaluating the efficacy of innovative drugs in melanomas. Human melanomas (*n* = 17) of different genotypes (mutated BRAF, NRAS, amplified cKIT and wild type) were successfully engrafted in mice then amplified by successive transplantations. The exhaustive characterization of patient-derived xenografts (PDX) at genomic level (transcriptomic and CGH arrays) revealed a similar distribution pattern of genetic abnormalities throughout the successive transplantations compared to the initial patient tumor, enabling their use for mutation-specific therapy strategies. The reproducibility of their spontaneous metastatic potential in mice was assessed in 8 models. These PDXs were used for the development of histoculture drug response assays (*ex vivo*) for the evaluation of innovative drug efficacy (BRAF and MEK inhibitors). The pharmacological effects of BRAF and MEK inhibitors were similar between PDX-derived histocultures and their corresponding PDX, on 2 models of BRAF and NRAS-mutated melanomas. These models constitute a validated, effective tool for preclinical investigation of new therapeutic agents, and improve therapeutic strategies in the treatment of metastatic melanoma.

## INTRODUCTION

With a 5-year survival rate under 10% with standard chemotherapies, metastatic melanoma has long been considered as disease with poor prognosis [1]. Melanomas are heterogeneous tumors, harboring distinct profiles of somatic mutations involved in tumorigenesis [2]. In addition to the traditional treatments based on chemotherapy, molecularly targeted drugs such as BRAF or MEK inhibitors have recently been developed and have shown clinical benefit in BRAF-mutated melanomas [3, 4]. Nevertheless, acquired resistance to these treatments occurred almost systematically [5]. Assessing the efficacy of other various targeted therapies could guide the therapeutic choice at the time of tumor relapse. Moreover in case of primary resistance, the development of personalized therapies could be critical to improve the prognosis of metastatic melanoma [6].

The development of personalized medicine requires preclinical models derived from patient tumors that reproduce their molecular characteristics. Tumor cell lines are widely used for preclinical studies, but after *in vivo* transplantation, they do not reflect the original structural and molecular characteristics representing human tumor heterogeneity, thus limiting their predictive value [7]. To overcome these limitations, patient-derived tumor xenograft (PDX) models have been established by engraftment of fresh tumor samples directly into immunodeficient mice [8, 9]. PDXs retain the molecular profiles, pathological characteristics and biomarker status of the original patient tumors, and are therefore relevant preclinical models to study response to treatments [9]. Although series of PDX models have been established [10-13], no study with genomic characterization and validation through the transplantations have yet been reported in cutaneous melanoma. In order to enable accurate drug testing, it is important to ensure that no significant genetic drift has occurred between the primary human tumor and the engrafted samples through the successive generations of mice. PDX models could be used for drug testing, enabling the identification of the most effective therapeutic regimen for each patient [14, 15].

A major limitation to the development of PDX models is the cost and time required for the maintenance of “live tumor banks” [14]. In addition, patients with poor life expectancy may not have enough time to benefit from PDX models. Histoculture drug response assay (HDRA) is an *ex vivo* assay used to assess the sensitivity of tumor cells to treatments, conducted with freshly removed patient tumors. By maintaining the three-dimensional architecture of native tissue, it retains the phenotypic characteristics of the original patient tumor and more correctly reflects the *in vivo* environment [16, 17]. In various solid tumors, inhibition rates observed with HDRA were predictive of clinical response to chemotherapy [17-19]. In prospective studies conducted with gastric and ovarian cancer patients, HDRA response correlated to patient survival [16, 20].

In this study, we report a preclinical strategy to assess tumor sensitivity to BRAF and MEK inhibitors, *in vivo* using PDX models, and *ex vivo* using histocultures derived from PDXs. We characterized a series of patient-derived melanoma xenografts transplanted serially in immunodeficient mice, with regard to their genomic and transcriptional profiles. We then studied response to targeted therapies in melanoma histocultures in comparison with their corresponding PDXs.

## RESULTS

### Melanoma engraftment rate is correlated with tumor aggressiveness and clinical outcome

A total of 54 melanoma samples obtained from primary tumors or metastases after surgery were freshly implanted subcutaneously into nude or RAG mice. Of the 54 tumors, 17 (31.5%) showed full xenograft development. The nude mice models produced a 28.6% stable take rate, whereas the RAG mice models had a take rate of 36.8%.

The clinical and pathological characteristics of the tumors, and their effect on the take rate of the corresponding xenografts, are summarized in Table 1. The melanomas were surgically removed from 54 patients with stage I (*N* = 7), stage II (*N* = 2), stage IIIA/B (*N* = 18), or stage IIIC/IV (*N* = 22) disease. The engraftment rate increased when the tumor samples were collected from lymph nodes or metastases *versus* primary sites, with engraftment rates of 50% and 37.5% *versus* 6.3%, respectively. The stage of the disease at the time of tissue banking affected the *in vivo* tumor engraftment, which increased to 54.5% with tumors derived from advanced or metastatic melanomas (stage IIIC/IV), *versus* 14.8% for resectable melanomas. Primary tumor parameters such as the Breslow index or ulceration were associated to *in vivo* tumor engraftment. The mutational status for *BRAF*, *NRAS* and *c-KIT* was assessed in the melanoma samples. Respectively 50%, 30%, 33% and 20% of *BRAF*-, *NRAS*-, *c-KIT*-mutated or wild type melanomas were successfully engrafted.

**Table 1:** Patient and tumor characteristics: distribution and corresponding tumor take rates in mice

Patient and tumor characteristics	Frequency in the study population, N (%)	Take rate, N (%) *
**Melanoma: histologic type**		
** – SSM**	22 (46.8)	9/22 (40.9)
** – Nodular**	10 (21.3)	4/10 (40.0)
** – ALM**	8 (17.0)	2/8 (28.6)
** – Dubreuilh**	4 (8.5)	0
** – Mucosal**	1 (2.1)	0
** – Unclassifiable**	2 (4.3)	0
** – NA**	7	2
**Breslow index, mm**		
** – <1 mm**	12 (26.1)	3/12 (25.0)
** – ≥ 1 mm**	34 (73.9)	12/34 (35.3)
** – NA**	8	2
**Ulceration**		
** – Yes**	21 (51.2)	11/21 (52.4)
** – No**	20 (48.8)	2/20 (10.0)
** – NA**	10	4/10
** – NR**	3	-
**AJCC staging at the time of tissue banking**		
** – Resectable**	27 (55.1)	4/27 (14.8)
** – Non resectable (IIIC/IV)**	22 (44.9)	12/22 (54.5)
** – NA**	5	1
**Mutational status**		
** – *BRAF* mutated**	18 (35.3)	9/18 (50.0)
** – *NRAS* mutated**	10 (19.6)	3/10 (30.0)
** – *KIT* mutated/amplified**	3 (5.9)	1/3 (33.3)
** – WT**	20 (39.2)	4/20 (20.0)
** – NA**	3	0
**Origin of tumor sample**		
** – Primary site**	16 (29.6)	1/16 (6.3)
** – Lymph node**	14 (25.9)	7/14 (50)
** – Metastatic site**	24 (44.4)	9/24 (37.5)
**Immunocompromised mice**		
** – Nude**	35 (64.8)	10/35 (28.6)
** – RAG**	19 (35.2)	7/19 (36.8)

* Tumor take rate at the first passage

AJCC, American Joint Committee on Cancer, ALM, acrolentiginous melanoma; mm, millimeters; NA, not available; NR, not relevant; SSM, superficial spreading melanoma

**Figure 1:**
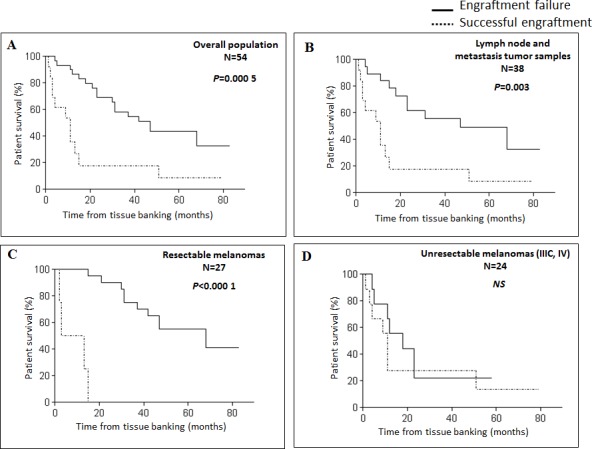
Xenograft take correlates with survival in melanoma patients Kaplan-Meier survival curves according to xenograft take for the overall population **A.**, patients with lymph node or metastasis tumor samples **B.**, patients with resectable melanomas **C.** or patients with advanced melanomas (stage IIIC/IV) **D.** NS, not significant.

To assess whether the engraftment efficiency reflected melanoma aggressiveness, we collected the clinical outcome of patients included in the study, with a mean follow-up of 15 months from tissue banking. Median survival was 31 months in the overall study population, and 11 months for the metastatic patients. Engraftment take in mice was predictive of shorter overall survival (OS) with a median of 11 months from tissue banking, *versus* 47 months in case of engraftment failure (*P* = 0.0005) (Figure 1A). Successful engraftment was also correlated to OS in the subsets of lymph node and metastasis tumor samples (*P* = 0.003) (Figure 1B). A significant association was found between OS and the engraftment rate for resectable melanoma-derived samples (*P* < 0.000 1) (Figure 1C). In the context of stage IIIC/IV melanoma, median OS was 6.5 months for patients with successfully engrafted tumors *versus* 15 months in case of failure (*P* not significant) (Figure 1D).

### Growth parameters of melanoma xenografts

The phenotypic and genotypic characteristics of the first 8 xenograft models were more precisely evaluated. The tumor graft latency, defined as the time from transplantation to a tumor size of 200 mm^3^, ranged from 10 to 36 days; the doubling time measured between 500 and 1 000 mm^3^ ranged from 3 to 11 days; the time to reach 1500 mm^3^ after initial transplantation was highly variable, with values ranging from 2.3 to 14.3 months. These parameters were recorded after the first transplantation (Table S1). The xenograft tumors grew faster after 5 transplantations than at the time of the first implantation (mean tumor growth rate: 37 vs. 12 mm^3^/day) (*P* < 0.003) (Figure S1A). The tumor growth was similar between the 2 models derived from the same patient, respectively from the metastatic lymph node and the primary tumor (Mel-X4 and Mel-X6) (mean tumor growth rate: 26 and 19 mm^3^/day, *P* = 0.3) (Figure S1B). A delayed time for PDX to reach 1500 mm^3^ >4 months was associated with improved OS for patients, with a median OS of 15 months vs. 2,5 months (*P* = 0,04) (Figure S2).

### Metastatic potential and the expression of invasion genes

**Figure 2:**
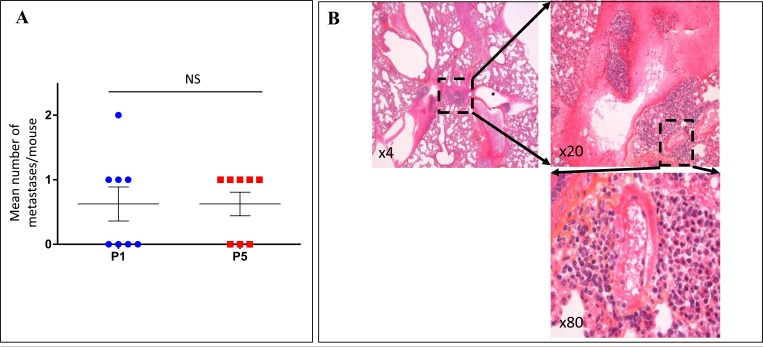

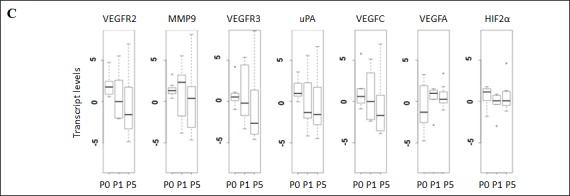

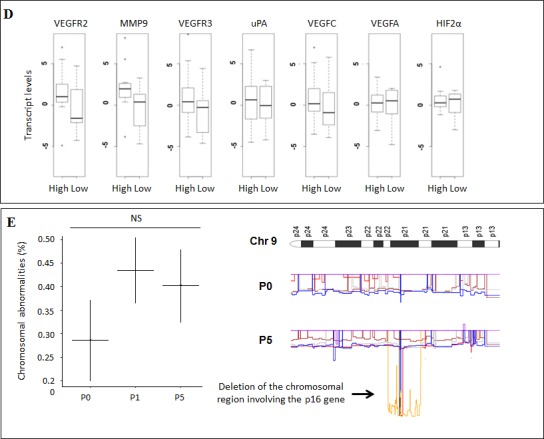
Xenografted melanoma tumors retained the metastatic potential and the expression profiles of invasion genes after successive transplantations in mice For the 8 characterized models of xenografted melanomas: **A.** Metastatic efficiency in the first and the fifth xenograft generations after successive transplantations. Metastatic efficiency is evaluated as the median number of metastases per mouse for each generation. Each plot represents the mean number of metastases for 3 mice derived from a same model (Mel-X1 to Mel-X8). Mean±SEM; NS, not significant; P1, first passage; P5, fifth passage. **B.** Hematoxylin-eosin (H&E) sections showing an example of lung metastasis on PDX mice. **C.** Variations of transcript levels with number of passages. Differences in the invasion gene expression expressed between corresponding xenografts after 1 or 5 passages (respectively P1 and P5), in comparison with original tumor samples (P0) are not statistically significant (*P* = 0.1). Gene expressions analyzed with qPCR are related to the β2-microglobulin gene expression at each generation (log-scale value). **D.** Variations of transcript levels with metastatic potential. Invasion gene expressions are not significantly different between xenografts at low and high metastatic potential (*P* = 0.1). Gene expressions analyzed with qPCR are related to the β2-microglobulin gene expression (log-scale value). **E.** Chromosomal abnormalities, in percentages, in the initial tumors (P0), first (P1) and fifth passages (P5); mean±SEM; NS, not significant. The deletion of the chromosomal region involving the *P16* gene in chromosome 9 is more frequently observed at P1 and P5 than at P0 (*P* < 0,05).

The ability to form distant metastases was followed through the successive transplant generations of the 8 characterized models. Four melanomas (Mel-X5, Mel-X6, Mel-X7, and Mel-X 8) metastasized widely, while the other 4 did not form multiple distant metastases (Table S1). Fifteen metastases at P1 and 16 metastases at P5 were formed, suggesting that the xenografts derived from the same metastasizing model tended to retain the ability to form distant metastasis between the first (P1) and the fifth passage (P5) (Figure 2A). Metastases were most commonly found in the lungs and the liver (Figure 2B). The 2 melanomas that formed lung or liver metastases in patients also metastasized to the lungs or liver in mice (respectively Mel-X1 for lung metastases and Mel-X4 and Mel-X6 for liver metastases) (Table S2).

In order to assess the molecular stability of the metastatic phenotype across serial transplantations, we investigated the transcriptional status of 7 genes involved in invasion and angiogenesis (*KDR* (*VEGFR-2)*, *FLT4* (*VEGFR-3*), *VEGFC, VEGFA*, *HIF-2α*, *PLAU* (*uPA*), *MMP9*) [21-23] and 3 genes involved in melanocytic differentiation (*MITF, TYR, MLANA* (*MART1*)) [24] using quantitative RT-PCR to compare xenografted tumors across serial transplantations. The human mRNA expression of the invasion genes was not significantly different between xenografts at P0, P1 and P5 *(P* = 0.1) (Figure 2C). The murine mRNA expression of the same genes was not modified between xenograft transplants (data not shown). The expression of human and mouse differentiation genes did not vary significantly between the passages (data not shown). These results were consistent with a stability of the metastasis phenotype after transplantation (Figure 2A).

Compared to the metastatic potential, only MMP-9 tended to be associated with the metastatic rate (*P* = 0.1) (Figure 2D). The murine mRNA expression of the same invasion and angiogenesis genes was not correlated to the metastatic potential (data not shown). In multivariate analysis no association was observed between the expression of angiogenesis and invasion genes and the metastatic rate.

### Evaluation of genetic stability through serial transplantation

Molecular and genetic characterization was performed before the serial transplants (P0), after the first (P1) and the fifth transplantations (P5) on the 8 engrafted tumors. To validate the concordance between serial transplantations, the xenografts were characterized for genetic parameters using the CGH array technique. In the CGH arrays the transplantation generations from each xenograft model clustered together, meaning that the first and the 5^th^ generations consistently shared the main alterations (Figure S3). The mean rate of chromosomal abnormalities did not significantly increase after the first passage (respectively 0.28 and 0.43%) and remained globally stable through 5 transplantations (0.40%) (*P* = 0.17) (Figure 2E). Nevertheless an alteration on chromosome 9, resulting in the deletion of the tumor suppressor gene *p16*, was more frequently observed in the first and fifth generations of xenografted tumors than in the initial tumors (*P* < 0.05). Only the Mel-X3 model expressed the *p16* deletion before xenografting, while 6 models were deleted after 5 transplantations (Figure 2E). Overall, our PDX models retained the molecular characteristics across numerous transplant generations, demonstrating an overall stability of the xenografted tumor genome.

### *In vivo* and *ex vivo* anti-tumor efficacy of targeted therapies

We next applied these stable PDX models for *ex vivo* drug evaluation using histoculture drug response assay. For this, 2 established PDXs harboring distinct mutational profiles (BRAF^V600E^ mutated Mel-XA and NRAS^G13R^ mutated Mel-XB) were used, both obtained from lymph node metastases after 4 transplantations. A BRAFi, vemurafenib, and a MEKi, pimasertib, currently used in the treatment of BRAF-mutated metastatic melanoma were evaluated in this way.

**Figure 3:**
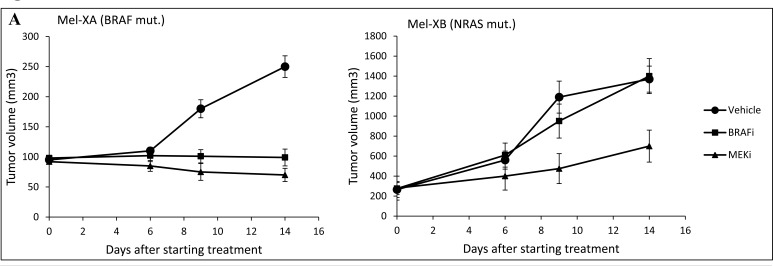

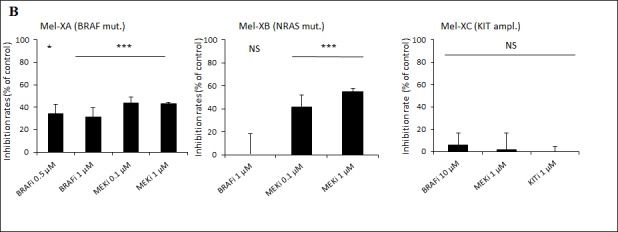

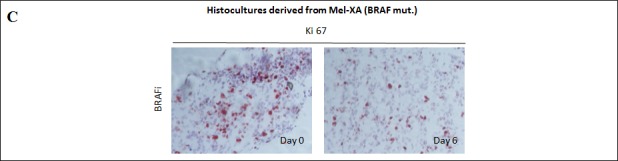
Comparative therapeutic experiments on patient-derived tumor xenografts (PDXs) and xenograft-derived histocultures **A.** Effect of BRAF or MEK inhibitors on tumor growth of PDXs. Growth of Mel-XA xenograft expressing the V600E BRAF mutation was inhibited by BRAF and MEK inhibitors (*P* < 10^−5^), while the tumor growth of Mel-XB harboring the G13R NRAS mutation was only inhibited by MEK inhibitor (*P* < 10^−4^). Controls were treated with PBS-DMSO 5%. Three mice per model were tested in each treatment and control group. **B.** Inhibition rates of BRAF and MEK inhibitors on Mel-XA and Mel-XB-derived histocultures determined by MTS assay, related to control treatment with DMSO). Mel-XC-derived histocultures was used as negative control. The results are representative of 3 independent experiments. Mean±SEM; NS, non significant; *, *P* < 0.05; ***, *P* < 0.00 1. **C.** Immunohistochemical staining with the Ki67 antibody in MEL-XA derived histocultures from the group treated with vemurafenib.

*In vivo* treatment of Mel-XA xenograft with either vemurafenib or pimasertib was effective, with tumor growth delay (TGD) >4 days for both treated groups. The tumor growth inhibition was significant with both treatments (*P* < 10^−5^), with tumor stabilization observed for vemurafenib treated group (ΔT/ΔC % = 1% at day 14) and pimasertib treated group (ΔT/ΔC % = −14% at day 14), with ΔT and ΔC being the evolution of mean tumor volume from day 0 respectively in the treated group and in the control group (Figure 3A). For Mel-XB, the primary tumor growth was significantly inhibited by pimasertib 9 days after starting treatment, with a TGD>6, while it was not sensitive to vemurafenib, with TGD = 1. The tumor growth inhibition expressed with the ΔT/ΔC ratio was 102% with vemurafenib (ns) and 38% with pimasertib (*P < 10^−4^*) at day 14 (Figure 3A).

Tumor tissues were freshly removed from these 2 PDXs to perform HDRA. PDXs were sliced, placed on gelatin-coated surface, and cultured for 6 days in presence of inhibitors or control. We examined the cytotoxicity of BRAFi (vemurafenib, 0.5 μM, 1 μM and 10 μM) or MEKi (pimasertib, 0.1μM and 1 μM) compared with DMSO as control through MTS assay (Figure 3B). Mel-XC, a PDX harboring a KIT amplification was used as a negative control. For the histocultures derived from BRAF-mutated Mel-XA, the average inhibition rates were 32.5 +- 8.6 % for BRAFi, and 43.5 +- 3.1 % for MEKi. The Ki67 level determined by immunohistochemistry in these sections of histocultures confirmed the inhibition of tumor cell proliferation by BRAFi (Figure 3C). For histocultures derived from RAS-mutated Mel-XB, the average inhibition rates were 48.3 +- 3.7 % with MEKi, whereas no effect was observed with BRAFi alone. There was no difference in inhibition rates between the 2 targeted therapies and the control treatment in the histoculture derived from the KIT-amplified tumor Mel-XC.

Taken together, we demonstrated that tumor inhibition in HDRA *ex vivo* was correlated with the growth inhibition observed in PDXs *in vivo* for 2 patients, with similar patterns of response for each drug tested.

## DISCUSSION

The aim of the present study was to develop a novel preclinical model to predict response to targeted therapies, based on PDX-derived histocultures. It requires establishing the stability of melanoma PDXs through serial transplantations prior to conducting therapeutic experiments.

We established a series of melanoma xenografts into immunocompromised mice, and achieved a successful engraftment rate of 31.5 %, which is in line with a study conducted on a series of uveal melanomas [10]. Metastatic lymph nodes had a higher take rate than metastases (mostly cutaneous), and primary tumors [10, 11]. The engraftment rate was correlated with validated poor prognosis factors in melanoma, such as increased Breslow index, ulceration or stage IIIC/IV metastatic melanoma, which represent the tumor aggressiveness. As in series of uveal melanoma or pancreas PDXs [10, 25], we noted a significant association between successful engraftment and poor survival.

PDX models could reflect the genetic diversity of patient tumors [10, 12, 13], which would enable development of personalized therapies [14]. A high level of concordance between the genetic alterations observed in patient tumors and those in their corresponding xenografts has been demonstrated in different cancer types [12]. Mice engrafted with human melanomas were shown to model the metastatic behavior of melanoma in patients [11]. By analyzing tumor genomic profile and metastatic efficiency in relation to the expression of invasion genes, we suggest that genomic and phenotypic characteristics and gene expression profiles were stable from primary human tumor through serial generations of xenografts in melanoma, as reported in other cancers [12, 26-28].

The similarity between xenograft generations (P1 and P5) was more marked than the similarity between the xenograft and the original tumor (P0 and P1). These findings suggest that the selection induced by the first tumor engraftment causes the non-significant genetic variability that we observed. There was a trend towards the enrichment of a deleted chromosomal region involving the *P16* gene over successive xenograft passages. *P*16 is a known tumor suppressor gene in melanoma. Loss of its expression occurs in 90% of metastatic melanomas and is essential to disease progression [29]. We hypothesized that this chromosomal aberration, acquired after xenografting, was related to intrinsic tumor progression rather than induced by a xenograft model-specific artifact. This observation has been reported in a preclinical model of sarcoma [30], and in a xenograft model of breast cancer in which most of the mutations detected in the primary tumor-derived xenograft were also observed in metastases, but not in the primary tumor [31]. These observations suggest that genomic progression may be similar during xenografting and the metastatic process.

PDXs are close to the human primary tumor and phenotypically and genetically stable through successive transplantations, thus being a relevant model to conduct pharmacological experiments. Some mechanisms of resistance to MAP kinase pathway-targeted therapies, which are now crucial in the setting of melanoma treatment, have been modeled in melanoma PDXs. Das Thakur et al demonstrated, in PDXs with acquired resistance to BRAF inhibitor, that intermittent instead of continuous dosing of BRAF inhibitor could prevent the emergent of resistant tumor cells [32].

Nevertheless the establishment of PDXs required 1 to 3 months for each generation prior to being used in preclinical experiments, which required 3 additional weeks of treatment. In order to take advantage of PDX models, which are close to patient tumors and enable amplification of small amounts of tissue specimen, we established PDX derived-histocultures enabling drug evaluation in 6 days. *Ex vivo* HDRA could assess tumor cell sensitivity to anticancer drugs in conditions similar to those *in vivo* and has previously shown a correlation rate of over 90% to clinical activity of chemotherapeutic agents [33, 34]. We applied this model to assess the sensitivity to MAP kinase pathway inhibitors. We conducted therapeutic experiments on 2 melanoma PDX models, *in vivo* and *ex vivo* using HDRA. HDRAs were performed using tumor tissues directly removed from PDXs, which avoid losing the characteristics of original tumors across culture pas­sages. MAP kinase pathway-targeted therapies were tested in accordance with the profile of mutations that the tumors harbored. In both models, the inhibition rates in histocultures were similar to the reduction in tumor growth in PDXs for each drug tested. Both HDRA and PDX models derived from the *BRAF*-mutated melanoma showed significant response to BRAF and MEK inhibitors, and those derived from the *NRAS-*mutated melanoma showed a significant response only to the MEK inhibitor pimasertib. These data are in line with the response profile to targeted therapies in melanoma [3, 4, 35].

The concordance of results between xenografts and histocultures in therapeutic experiments has been reported in a pancreatic cancer model [36]. We suggest here that HDRAs are also feasible with tumors removed from PDXs. HDRAs have the advantage of providing information about sensitivity to numerous therapeutic agents within a short time frame, while PDX enables cancer tissue propagation *in vivo*, resulting in more tissue material available for therapeutic experiments. HDRAs can be performed as many times as needed as long as PDX-engrafted mice are available in a “live tumor bank”. This method could contribute to the therapeutic choices in the management of metastatic melanomas.

Nevertheless, there are some limitations associated with this method that need to be stressed. First, because of the immunocompromised nature of the mice, therapeutic experiments based on immunotherapeutic agents cannot be conducted. Second, the engraftment efficiency varies significantly between patient tumors, and is correlated to tumor aggressiveness. In case of engraftment failure, the less aggressive tumors would therefore not be studied. The use of other models of immunocompromised mice, such as NOG mice, which have recently demonstrated a better take rate for melanoma xenograft, could help to overcome this issue [13]. However the level of immunosuppression in mice, in particular the presence of NK cells, may influence the tumorigenic potential of melanoma cell subpopulations [37].

To conclude, our study demonstrates that melanoma tumor response to targeted therapies can be assessed in histocultures as well as in their corresponding PDX models, which retain genomic, transcriptional and phenotypic characteristics of the original patient tumors. This preliminary study reports the consistency of both preclinical melanoma cancer models. These models could contribute to developing personalized medicine, by integrating their predictive value into therapeutic strategies.

## MATERIALS AND METHODS

### Patient tumor samples and establishment of melanoma xenografts

Fifty-four melanoma specimens were obtained from patients with histologically proven melanoma disease, at the time of surgery between 2009 and 2013, after informed patient consent. For each specimen, fragments of 5 mm^3^ were freshly grafted subcutaneously into the interscapular fat pad of three 8- to 12-week-old female nude (Janvier) or RAG (provided by local Animal Facility, IUH) anesthetized mice. The mice were maintained in specific pathogen-free animal housing, according to French regulations. Failure of engraftment was considered if no tumor was palpable after 12 months. They were euthanized by cervical dislocation when their tumor reached a volume of 1500 mm^3^. Patient and tumor characteristics were collected to study factors of *in vivo* engraftment: age, gender, histological type of melanoma, Breslow index, ulceration, mutational status for *BRAF*, *NRAS* and *c-KIT*, origin of tumor sample, AJCC staging (American Joint Committee on Cancer) at the time of surgery, and overall survival. Overall survival was measured as the time from the date of tissue banking to the date of patient death.

### Mutation analysis

DNA was extracted using the DNeasy Blood and Tissue Kit (Qiagen, France). DNA was quantitated using a NanoDrop ND-1000 spectrophotometer (NanoDrop Technologies, USA).

*BRAF*, *NRAS* and c-*KIT* mutation analyses were performed as previously described [38, 39] using routine methods used in our hospital. Briefly, molecular genotyping was performed on a LightCycler 480 (Roche, France) using a *BRAF*^V600E^ allele specific discrimination assay and an exons 2 and 3 *NRAS* specific High Resolution Melting assay, both followed by a bidirectional sequencing on an ABI 3130 XL automated sequencer (Applied Bioscience, France). *c- Kit* exons 11, 13, 17 and 18 mutation analyses were performed as previously described [40] using a PCR amplification followed by Sanger sequencing on an ABI 3130 XL.

### *In vivo* tumor growth and metastatic potential

The 8 first tumor xenograft models were more precisely characterized. Xenografts appeared at the graft site 3 to 11 months after grafting in nude mice. They were subsequently transplanted from mouse to mouse for 5 consecutive transfers, with 3 mice per passage for each model. Tumor samples were stored frozen in liquid nitrogen for genomic analyses, or formalin-fixed for immunohistochemical analyses.

*In vivo* tumor growth was determined by the tumor growth latency, defined as the time to reach a tumor volume of 200 mm^3^, the doubling time (between 500 and 1000 mm^3^) and the time to reach 1500 mm^3^. Measurements of tumor size were calculated twice weekly. Two-dimensional measurements were taken with a caliper and tumor volume (V) was calculated using the following formula to calculate the volume of an ellipsoid: V = L*l^2^/2, where L is the longest diameter, l the shortest one. Mean and standard error of the mean (SEM) of the volume were calculated for each model and growth curves were established as a function of time.

After euthanizing, the tissue samples from lymph nodes and organs were collected. The presence of metastases, defined as cells that are like to the xenografted tumors, was assessed by systematic microscopic analysis by two pathologists (MV, AJ). Metastatic efficiency was defined as high or low on the basis of the number of sites of metastases related to the number of mice, respectively more or less than 50%.

### Array-based comparative genomic hybridization (CGH)

Genomic DNA was isolated from xenografts before the serial transplants (P0), after the first (P1) and the fifth transplantations (P5), using DNeasy extraction kit (Qiagen, France) according to the manufacturer's instructions. The percentage of mouse and human component was determined by quantitative PCR (qPCR) using species-specific primers. CGH labeling and hybridization were performed using high-density 244K arrays (Agilent, France). Sample DNAs were labeled with Cy5-dUTP and Cy3-dUTP, respectively. Labeled products were purified with Microcon YM-30 filters (Millipore, Billerica, MA). Arrays were scanned with an Agilent DNA Microarray Scanner (G2565BA). Log2 ratios were determined with Agilent Feature Extraction software (v9.1.3.1). The global quality of the individual microarrays was validated against the quality metrics (QCmetrics) provided in this software. Results were analyzed with Agilent's CGH Analytics v3.5 software. Copy number aberrations (CNA) were detected using the Aberration Detection Method algorithm 2 (ADM-2) with a threshold of 6. At least two contiguous suprathreshold probes were required to define a chromosomal abnormality. Percent of chromosomal abnormalities was defined as the number of CNA upon total number of probes.

### Evaluation of gene expression implied in invasion, angiogenesis and differentiation

The expression of tumor-specific antigens was assessed by real-time quantitative reverse transcription-PCR on RNA extracted from tumor xenograft samples. Total RNA extraction and cDNA synthesis were performed from 1 μg total RNA (Qiagen, France). KDR (VEGFR2), FLT4 (VEGFR3), VEGFC, VEGFA, MMP-9, HIF-2α and PLAU (uPA) transcripts involved in invasion and angiogenesis [21-23], and MITF, TYR and MLANA (MART1) involved in melanocytic differentiation [24] were quantified. Specific probes and primers were used to evaluate the expression of human or mouse transcripts. Transcript levels were measured using Perfect Master Mix-Probe (AnyGenes, France) on LightCycler 480 (Roche, France) according to the manufacturer's protocol. The transcript levels were normalized to the housekeeping β2-microglobulin transcripts. Differences between different groups (P0, P1, P5) were assessed by multivariate regression (glm command in R.3.01 software, Foundation for Statistical Computing, Vienna, Austria).

### Evaluation of targeted therapies on patient-derived xenografts

Two PDX models harboring distinct mutations were selected for therapeutic experiments, as they were expected to have different profile of response to BRAF inhibitor [41]. They were obtained from lymph node metastases as described in Section 2.1 after 4 transplantations. Mel-XA expressed the BRAF^V600E^ mutation and Mel-XB the NRAS^G13R^ mutation. Three mice per model were tested in each treatment and control group. Targeted therapies were based on vemurafenib (Roche, France) as a BRAFi (BRAF inhibitor), and pimasertib (Merck-Serono, Germany), as a MEKi (MEK inhibitor), which are becoming standard treatments for BRAF-mutated melanomas. Treatment started when the average tumor size was approximately 300 mm^3^ and was administered by oral gavage twice daily (vemurafenib, 50 mg/kg/day or pimasertib, 30 mg/kg/day). The control group was treated twice daily with PBS-DMSO 5%. During treatment, tumor growth was determined twice weekly. Antitumor activity of the compounds was evaluated by calculating the tumor growth delay (TGD) and tumor growth inhibition ΔT/ΔC ratio. TGD was defined as the time difference (in days) between treatment and control groups to grow to 200 mm^3^ or 1000 mm^3^, respectively for Mel-XA and Mel-XB, compared with the untreated control. The ΔT/ΔC ratio was calculated as the ratio of the mean tumor volume for the treated vs. control group. ΔT was calculated as (mean tumor volume at day *X-* mean tumor volume at day 0) in the treated group, and ΔC as (mean tumor volume at day *X-* mean tumor volume at day *0*) in the control group, and ΔT/ΔC % as (ΔT/ΔC × 100).

### Xenograft-derived histoculture drug reaction assay (HDRA)

HDRA procedures were performed from 3 PDXs obtained from lymph node metastases after 4 transplantations for *in vivo* therapeutic experiments. Two PDXs harboring BRAF and NRAS mutation were tested (Mel-XA and Mel-XB), and a PDX harboring KIT amplification was used as a negative control (Mel-XC). Briefly, mice were euthanized when their tumor reached a volume of ≈1 cm^3^. A tumor fragment was freshly removed from PDX, washed and sliced. Sections were placed on gelatin-coated surfaces (Sigma-Aldrich, USA) in 24-well microplates, in triplicate. The histocultures were incubated for 6 days at 37°C (in a humidified atmosphere containing 95% air - 5% CO2) in presence of drugs or DMSO as control, dissolved with DMEM medium (Gibco, Invitrogen, USA) containing 20% fetal calf serum. Concentrations of vemurafenib were 0,5 μM, 1 μM and 10 μM, and those of pimasertib were 0,1 μM and 1 μM, on the basis of previous therapeutic assessments performed in our laboratory. After incubation, MTS (Promega, USA) was added and absorbance was read at 540 nm (BMG Labtech, UK). The inhibition rate was calculated by using the following formula: inhibition rate (%) = (1 - T/C) * 100, where T and C are respectively the mean absorbance of the treated and the control tumor. An inhibition rate over 30% was considered significant. For histocultures, slides were stained for Ki67 (M7240, Dako, Denmark) to assess the proliferation of tumor cells.

### Statistical analysis

Values were expressed as means ± standard deviation.

Differences in survival of the different groups were evaluated with a log-rank test. In the histoculture drug reaction assays, differences in inhibition rates between groups were evaluated using the t-test. All statistical tests were two-sided, and a p-value of less than 0.05 was considered as statistically significant. Statistical analyses were performed using Prism 6 (GraphPad Software Inc, La Jolla, CA) and R 3.01 software (Foundation for Statistical Computing, Vienna, Austria) (JPF). Tumor growth curves were compared using the grofit R package.

## SUPPLEMENTARY MATERIAL TABLES AND FIGURES



## Abreviations

HDRA: histoculture drug response assay
OS: overall survival
PDX: patient derived tumor xenograft.
