# Correction: Validation of a preclinical model for assessment of drug efficacy in melanoma

**DOI:** 10.18632/oncotarget.10822

**Published:** 2016-07-25

**Authors:** Delyon Julie, Varna Mariana, Jean-Paul Feugeas, Aurélie Sadoux, Saliha Yahiaoui, Marie-Pierre Podgorniak, Geoffroy Leclert, Sarra Mazouz Dorval, Nicolas Dumaz, Anne Janin, Samia Mourah, Céleste Lebbé

Present: Due to an error during submission of the revised manuscript, Dr. Jean Soulier's name was omitted from the author list.

Corrected: Correct author list for this article is available below: Authors sincerely apologize for this oversight.

Original article: Oncotarget. 2016; 7(11): 13069-81. doi: 10.18632/oncotarget.7541.

Julie Delyon^1,2,3^, Mariana Varna^4,5,6^, Jean-Paul Feugeas^3,7^, Aurélie Sadoux^1^, Saliha Yahiaoui^1^, Marie-Pierre Podgorniak^8^, Geoffroy Leclert^1^, Sarra Mazouz Dorval^3,9^, Nicolas Dumaz^1,3^, Jean Soulier^10,11^, Anne Janin^4,5,12^, Samia Mourah^1,3,8,*^ and Céleste Lebbé^1,2,3,*^

^1^ INSERM UMR_S976, Paris, F-75010, France

^2^ AP-HP, Hôpital Saint-Louis, Department of Dermatology, Paris, F-75010, France

^3^ Université Paris-Diderot, Sorbonne Paris Cité, Paris, F-75013, France

^4^ INSERM UMR_S1165, Paris, F-75010, France

^5^ Université Paris-Diderot, Department of Pathology, UMR_S1165, Paris, F-75010, France

^6^ UMR CNRS 8612, Institut Galien-UFR de Pharmacie, Université de Paris-Sud, Châtenay-Malabry, F-92290, France

^7^ INSERM UMR_1137, Paris, F-75010, France

^8^ AP-HP, Hôpital Saint-Louis, Laboratoire de Pharmacologie Biologique, Paris, F-75010, France

^9^ AP-HP, Hôpital Saint-Louis, Department of Plastic, Reconstructive and Esthetic Surgery, Paris, F-75010, France

^10^ AP-HP, Hôpital Saint-Louis, Department of Biological Hematology, Paris, F-75010, France

^11^ Institute of Hematology (IUH), University Paris-Diderot, Paris, F-75010, France

^12^ AP-HP, Hôpital Saint-Louis, Department of Pathology, Paris, F-75010, France

^*^ These authors have contributed equally to this work

