# Supplementary material for: Validation of a preclinical model for assessment of drug efficacy in melanoma
Source: Oncotarget. 2016 Feb 20;7(11):13069–81. doi: 10.18632/oncotarget.7541 (PMC4914342; doi:10.18632/oncotarget.7541)
Supplement: Supplementary file 1 [file oncotarget-07-13069-s001.pdf]

Validation of a preclinical model for assessment of drug efficacy in melanoma

Supplementary Material

Table S1: Tumor growth parameters and metastatic potential of 8 characterized xenografted models

Tumor xenograft	Origin of tumor sample	Mutational status	Tumor growth latency¶ (days)	Doubling time (days)	Time to reach 1500 mm^3 (months)
Mel-X1	Lymph node	WT	35.9	5.1	11.5
Mel-X2	Metastasis	BRAF mutated	10	3	1.5
Mel-X3	Lymph node	BRAF mutated	22.5	11	2.9
Mel-X4°	Lymph node	BRAF mutated	26.3	10.7	2.8
Mel-X5	Metastasis	WT	NA	NA	14.3
Mel-X6°	Primary site	BRAF mutated	21.3	7	3.3
Mel-X7	Metastasis	BRAF mutated	NA	NA	NA
Mel-X8	Lymph node	BRAF mutated	27.6	6	7

The parameters were recorded after the 1st transplantation.

NA, Not available; WT, wild type for the 3 genes analyzed (*BRAF, NRAS, c-KIT*)

¶ Mean time to a tumor size of 200 mm^3

° Tumors derived from the same patient

Table S2: Sites of metastasis and metastatic potential in xenografted mice

Tumor xenograft	Sites of metastasis									Metastatic rate *
	Lymph nodes	Lungs	Liver	Brain	Sternum	Kidney	Spleen	Ovary	Heart	
**Mel-X1**	0	4	1	0	0	0	0	0	0	Low
**Mel-X2**	0	2	2	0	2	0	0	0	0	
**Mel-X3**	0	0	1	0	0	0	0	0	0	
**Mel-X4°**	0	1	1	0	0	0	0	0	0	
**Mel-X5**	0	12	0	0	0	3	0	0	0	High
**Mel-X6°**	0	3	4	0	0	0	0	0	0	
**Mel-X7**	0	7	6	0	1	0	0	0	0	
**Mel-X8**	0	4	1	1	0	2	1	0	0	

The metastases were assessed by systematic histological evaluation. The metastatic potential was defined as the number of metastases related to the number of mice for each model.

* Number of metastases related to the number of mice.

° Tumours derived from the same patient

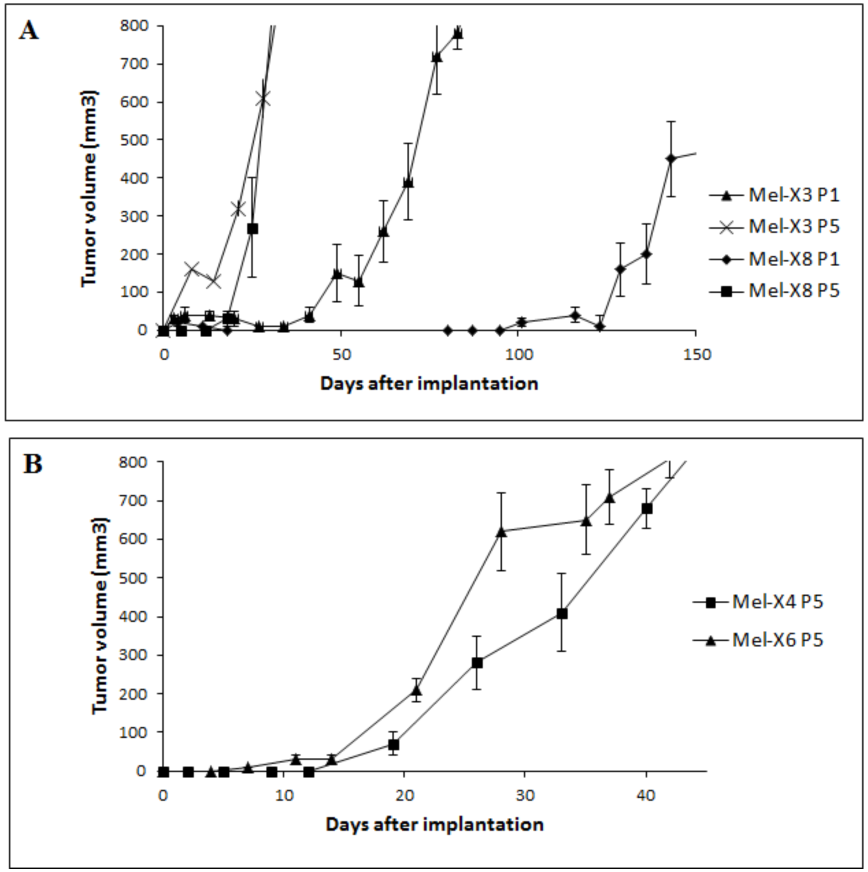

Figure S1: Spontaneous tumor growth of patient-derived tumor xenograft.

(A) Tumor growth curves of 2 representative xenografts (Mel-X3 and Mel-X8, respectively with low and high metastatic rate) at the first and the fifth passages. Xenografted tumors at P5 grew faster than xenografted tumors at P1 (mean tumor growth rate: 37 vs. 12 mm^3/day) (P=0.003).(B) Similar tumor growth of 2 models obtained from the same patient, respectively from the metastatic lymph node and the primary tumor (Mel-X4 and Mel-X6) (mean tumor growth rate: respectively 26 and 19 mm^3/day, P=0.3).

The results are representative of 3 independent experiments. Mean±SEM.

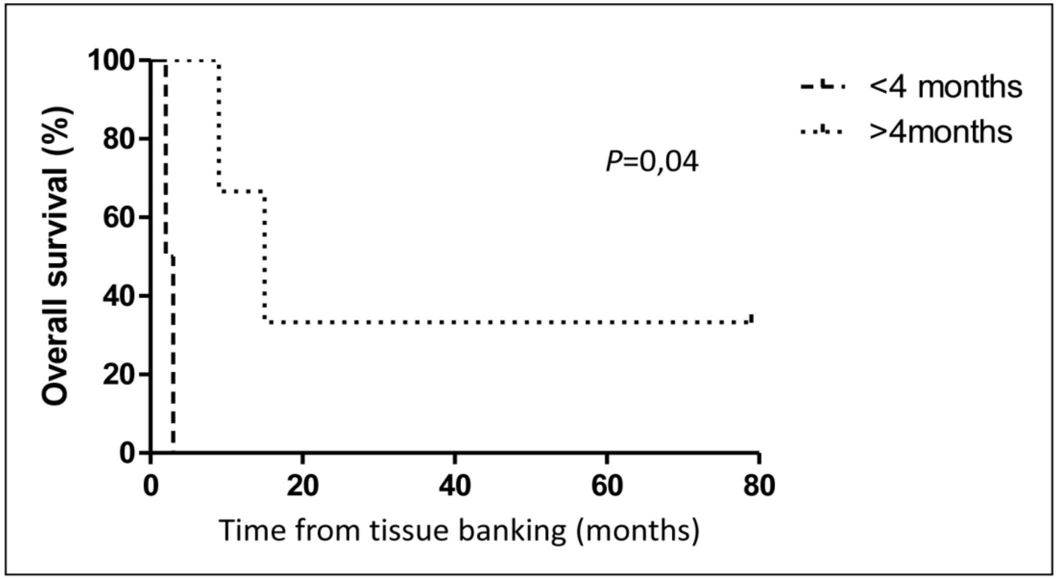

Figure S2: Tumor growth of PDX correlates with survival in melanoma patients.

Kaplan-Meier survival curves according to the time to reach 1500 mm^3 (cut-off, 4 months) for 7 melanoma PDXs.

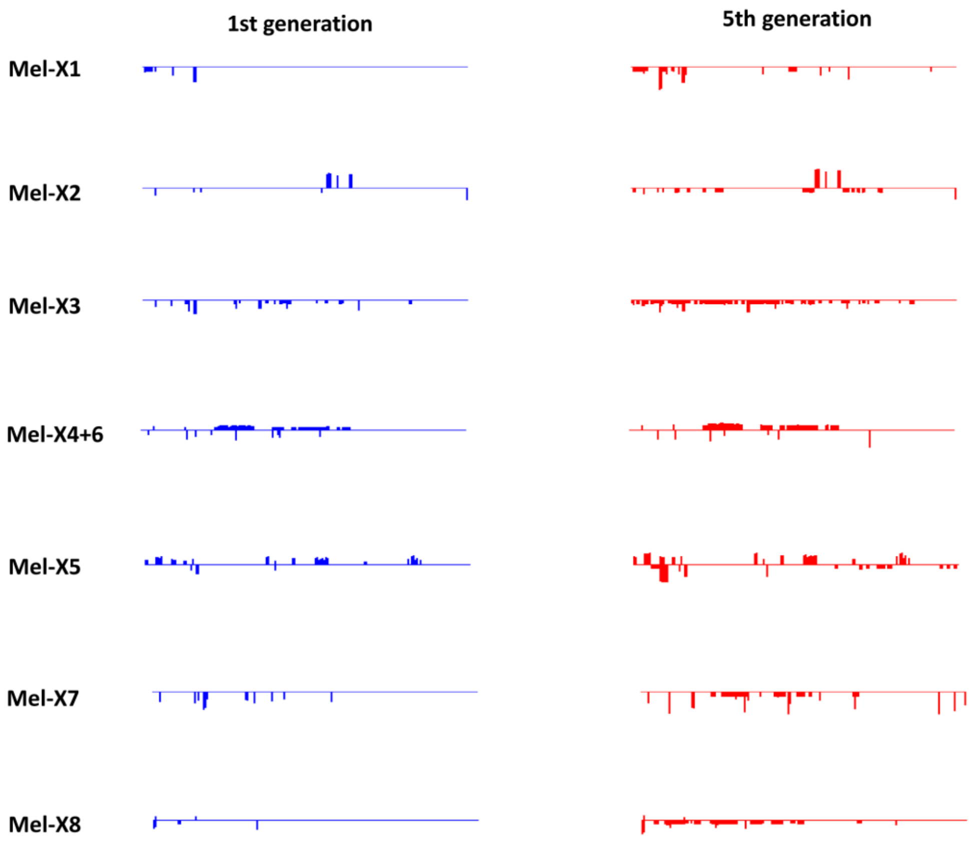

Figure S3: Tumor array CGH profiles of 8 xenografts before and after serial passages in mice. Loss, gain or amplification of chromosomal material compared with normal DNA are plotted on the y-axis and aligned along the x-axis in chromosomal order.
